# P60 A case of autoimmune inflammatory myositis in a patient with SLC29A3 spectrum disorder

**DOI:** 10.1093/rap/rkac067.060

**Published:** 2022-09-28

**Authors:** Adam Gittins, Eslam Al-Abadi

**Affiliations:** Birmingham Children's Hospital, Birmingham, United Kingdom; Birmingham Children's Hospital, Birmingham, United Kingdom

## Abstract

**Introduction/Background:**

The SLC29A3 gene encodes the equilibrative nucleoside transporter hENT3. SLC29A3 spectrum disorder, an autosomal recessive disorder caused by mutations in this gene, is a rare multisystem disorder resulting in cutaneous manifestations (hyperpigmented, hypertrichotic and indurated patches of skin) and a variety of systemic features, with variable phenotypic expression. Along this spectrum are H syndrome and pigmented hypertrichotic dermatosis with insulin-dependent diabetes (PHID) syndrome. The majority of cases reported in the literature have been in patients of Arab descent. We report a case of a patient with SLC29A3 mutation who later developed features of autoimmune disease and myositis.

**Description/Method:**

An 11-year-old girl with an established diagnosis of SLC29A3 mutation, dysosteosclerosis, camptodactyly and type 1 diabetes mellitus presented with a three-week history of intermittent fever, myalgia, muscle weakness and cervical lymphadenopathy. She had a non-specific rash over her eyelids, interphalangeal joints of both hands, torso and limbs. Her movements were restricted due to myalgia. She had no evidence of arthritis. Her creatine kinase was elevated (>1400 units/litre) and MRI of her thighs revealed evidence of myositis not akin to Juvenile Dermatomyositis distribution. An extensive investigation screen showed no evidence of infections. The bone marrow was hypocellular but there was no evidence of malignancy or histiocytosis. An autoinflammatory disease was suspected and the patient was treated with three pulses of IV methylprednisolone, to which she initially responded well but soon relapsed. Anakinra was trialled but this failed as the patient’s daily fevers persisted and her muscle weakness continued to worsen. Methotrexate was commenced and did not result in the desired reduction in prednisolone dose, so the patient was started on 4-weekly infliximab infusions. Following commencement of infliximab, the patient’s fever and rash resolved; her energy levels and muscle power improved and her muscle enzymes began to normalise. Repeat MRI showed her myositis was in remission. Infliximab was stopped after 13 doses due to side effects at the end of the infusions (tachycardia, hypertension and pyrexia). In addition, it was found that she had developed antibodies against infliximab and measured serum drug levels were low. Methotrexate was eventually stopped. The patient is now off all rheumatological treatment (as of February 2019) and she has not had reactivation of any of her symptoms.

**Discussion/Results:**

The patient we report had insulin-dependent diabetes mellitus, dysosteosclerosis and camptodactyly, three systemic features associated with SLC29A3 mutation. She presented concomitantly with signs of autoinflammatory disease and myositis. While up to 25% of patients with SLC29A3 mutation develop autoinflammatory features, to our knowledge, this is the first reported case of myositis in a patient with this mutation. Both the inflammatory features and myositis resolved following treatment with infliximab infusions. A number of case reports have described successful treatment of inflammation in SLC29A3 mutation with IL-6 inhibitors such as tocilizumab. The complete remission demonstrated in this case following infliximab infusions points to TNF-α inhibition as another potential therapeutic target. It is also interesting to note that our patient is now off all rheumatological treatment, which contrasts with other case reports in the literature where the patients have typically required maintenance immunomodulation therapy to remain asymptomatic. This case may therefore represent a new clinical subtype of SLC29A3 mutation with an autoimmune inflammatory myositis, rather than autoinflammatory as initially suspected, that carries a better prognosis. Further case reports of myositis in SLC29A3 mutation would help to build a clearer picture of its clinical presentation, prognosis and response to biologic therapies.

**Key learning points/Conclusion:**

1. SLC29A3 spectrum disorder is a rare multisystem disorder that has only recently been recognised. By presenting this case report we hope to increase awareness of this condition and network with other clinicians who have encountered inflammatory disease associated with SLC29A3 mutation, either in their clinical practice or research.

2. Up to a quarter of patients with SLC29A3 mutation present with autoinflammatory disease.

3. We report on the first case of autoimmune inflammatory myositis in a patient with SLC29A3 mutation, to our knowledge.

4. Our patient demonstrated complete remission of her inflammatory disease following treatment with infliximab, which may represent a new clinical subtype of SLC29A3 mutation with an autoimmune, rather than autoinflammatory, myositis that carries a better prognosis.

